# Exploring the Cognitive and Behavioral Risks and Maintenance Factors of Hikikomori: Protocol for an Ecological Momentary Assessment Study

**DOI:** 10.2196/81384

**Published:** 2026-02-17

**Authors:** Alexander MacLellan, Keisuke Takano

**Affiliations:** 1 Human Informatics and Interaction Research Institute, National Institute of Advanced Industrial Science and Technology, 1-1-1 Higashi, Tsukuba, 305-8566, Japan, 81 029-861-6001

**Keywords:** hikikomori, ecological momentary analysis, social withdrawal, social anxiety, internet addiction

## Abstract

**Background:**

Hikikomori is a state of social withdrawal first identified in Japan and is gaining interest globally. Classically, hikikomori is described as a state of isolation within one’s home, though recent conceptualizations have proposed a continuum of severity. Hikikomori frequently shares symptoms with depression, social anxiety, autism, and schizophrenia, as well as internet and gaming disorders. Clinical case studies and cross-sectional studies suggest that dysfunctional emotion regulation, familial support, and internet behaviors are proposed to contribute to the onset and maintenance of a withdrawn state, though they have not been explored longitudinally.

**Objective:**

This study aims to investigate affective, behavioral, and cognitive correlates of hikikomori symptoms, and how daily mood, social enjoyment, familial support, and internet usage may maintain a socially withdrawn state.

**Methods:**

A minimum of 84 participants aged between 18 and 60 years will complete self-report measures of hikikomori symptoms, internet addiction, depression, anxiety, autism, and fear of offending others before participating in 14 days of ecological momentary assessment surveys. Surveys will be delivered 5 times per day from 8 AM to 10 PM, measuring mood, internet behavior, familial relationships, social interaction frequency, anticipatory and consummatory enjoyment, sleep quality, and physical activity. Participants will repeat the self-report measure of hikikomori symptoms postmonitoring period.

**Results:**

Recruitment began in November 21, 2025. Data collection and analysis are scheduled to be completed by summer 2026, with the results also scheduled to be available by the end of summer 2026. Correlation and multiple regression analyses will investigate whether internet addiction, social anxiety, expressive suppression, fear of offending others, daily mood, internet use, social enjoyment, and familial support predict hikikomori symptoms. Time-lagged network analyses will explore the temporal dynamics of these relationships, and how these differ in those with high and low levels of hikikomori symptoms. Finally, time-lagged logistic regressions will explore which factors predict future social behavior.

**Conclusions:**

This study will be the first to investigate currently proposed mechanisms underlying hikikomori, while also exploring the time-varying relationships between affect and social behavior. The results will provide initial evidence for factors that predict hikikomori symptoms, explore candidate mechanisms underlying hikikomori, and identify potential maintenance factors as targets for intervention.

## Introduction

### Background

Hikikomori is a condition characterized by persistent isolation in one’s home alongside functional impairment and distress associated with this isolation [1]. First recognized and examined in Japan, hikikomori is now recognized globally [2]. The isolation is initially voluntary, similar to psychiatric disorders such as autism [3], who may find relief in their withdrawal from social situations; or in anxiety and depression [1], who may experience distress or apathy regarding socializing. However, there are some individuals who socially withdraw and yet do not present with any comorbid psychiatric disorders, raising questions over whether hikikomori relates to a distinct state or a transdiagnostic phenomenon [4]. Additionally, while classifying an individual as hikikomori has primarily been determined by the length and severity of their social withdrawal (eg, for 6 months), there is growing recognition of the condition existing on a spectrum of severity [1]. To date, much research has proposed onset and maintenance factors from clinical case reports [5] and cross-sectional studies [6]. Affect dynamics [1], social reward processing [7], internet behaviors [8], familial relationships [9], and physical health [10] have all been identified, though little has investigated the time-varying nature of these relationships and whether they predict social withdrawal tendencies in daily life.

Hikikomori shares patterns of disordered affect and emotion regulation with depression, social anxiety, autism, and schizophrenia [1]. Low mood and anhedonia are frequently present in hikikomori patients [1], leading to questions of whether hikikomori may be a subtype or expression of depression [1,2]. Additionally, hikikomori patients often express anger or display higher levels of irritability [5,11]. High irritability and expressions of anger are linked with impaired social outcomes [12], social anxiety [13], and well-being [14]. While sustained negative affect and distress are hallmarks of depression and anxiety and are often examined as traits, emotions are not fixed states and variation is expected. Intensity of emotional change, stability of the emotional state, and how quickly an emotion returns to baseline after a deviation are all predictive of psychological well-being [15,16]. Together, these patterns of emotional dynamics are found to be related to mental health and well-being, with less variable and more stable patterns, both in the frequency of change and the speed of return to baseline, found to be associated with better mental health [16]. This suggests that negative affect and emotional instability play a key role in the onset and maintenance of hikikomori. Differences in emotion regulation styles may underlie these affective variations, with adaptive strategies such as cognitive reappraisal found to be associated with positive affect, while maladaptive strategies such as rumination and suppression are associated with negative affect [17], as well as social anxiety and depression [18].

Equally, anhedonia may play a key role in the onset or maintenance of a socially withdrawn state. In populations with depression, anhedonia is linked with social isolation [19], which may be precipitated by a reduced generation of anticipatory enjoyment [7,20]. Learning from rewarding outcomes (eg, an enjoyable conversation) is also found to be impaired in depression [21] and social anxiety [22], suggesting that social withdrawal may partially result from reduced anticipatory enjoyment, failing to generate motivation to act, or failure to learn from a positive experience preventing an adaptive increase in anticipatory enjoyment at the next occasion. Other forms of communication, such as via the internet, may be more accessible in this case. Taken together, research from populations with both depression and social anxiety suggests that affect, including emotional variability and stability, and reward processing are implicated in social withdrawal. However, it is unclear how these factors relate to hikikomori symptoms and episodes of social withdrawal.

Aside from social withdrawal, behavioral hallmarks of hikikomori include problematic smartphone and internet use [8,23], excessively playing video games [24], a reliance on familial support [1], a more sedentary lifestyle, and poorer sleep quality [10]. Problematic internet use may allow and support a socially withdrawn state by replacing face-to-face connections with virtual interactions. While virtual interactions provide social contact with less risk of stress or challenge [23], this may also prevent individuals from challenging negative beliefs around in-person communication or developing positive resilience and coping strategies. Additionally, given the widespread availability of internet access, it is possible for individuals to work and manage their affairs solely online, again enabling a socially withdrawn state. Previous research has found links between problematic internet use, anhedonia, and well-being [25,26] and therefore is often a target both for research and intervention. Excessive gaming is also commonly researched as either a subtype of internet use or in isolation. Gaming may fill similar roles to internet use, namely as a coping mechanism, and equally may be a factor in the maintenance of social withdrawal [8,24].

Familial relationships are frequently discussed as a risk factor for a hikikomori condition [1]. Dysfunctional family relationships are common in observed cases of hikikomori [27], with both parental abuse [28] and overindulgence [9] linked to the socially withdrawn condition. Familial support may play a critical logistical role in the maintenance [9] and recovery [29] of hikikomori, though how this interacts with other variables is uncertain [9]. It may be that supportive actions (providing food and economic support), though initially made in the best interests of the patient, become maintenance factors that are difficult for the family to stop once started. Conversely, behaviors to challenge the withdrawn condition may also adversely affect hikikomori [28]. Therefore, uncovering how familial behaviors affect social withdrawal and interact with other factors is required.

Finally, sleep patterns, sleep quality, and physical activity may also play a role. Cases of hikikomori often note disordered sleeping patterns [10,30], such as primarily waking at night and sleeping during the day [28]. These patterns may have originated as a way to manage anxiety around other people, though as with defaulting to virtual communication, this may also increase feelings of disconnection and isolation [1]. When combined with a preference for virtual communication and isolation, the sedentary lifestyle noted in hikikomori samples is unsurprising and may contribute to poorer physical health [10].

### The Study

Taken together, hikikomori describes a condition that shares symptoms, cognitive profiles, and behavioral profiles with several mental health disorders [1]. While many researchers have proposed maintenance factors, to date, there has been little investigating how observed affective, cognitive, or behavioral factors influence symptoms, social motivation, and social withdrawal over time.

Ecological momentary assessment (EMA) can elucidate the time-varying nature of these relationships by repeatedly sampling daily mood and behaviors. EMA has previously been used to investigate social withdrawal and depressive symptoms [31], for example, previous studies have found that participants with clinical depression exhibit reduced anticipatory and consummatory enjoyment [32]. EMA therefore offers an opportunity to identify which daily behaviors and mood patterns are indicative of a risk of hikikomori symptoms and social withdrawal. Additionally, the EMA method allows us to investigate proposed factors and behaviors with high ecological validity given the measurements are occurring during the participant’s daily life.

This study will investigate affective, behavioral, and cognitive correlates of hikikomori symptoms, and how familial support and internet usage may maintain a socially withdrawn state. Over 2 weeks, participants will be prompted to complete 5 EMA surveys per day, with questions asking about their mood, internet activity, familial interactions, and social behavior. Surveys will be delivered every 3 hours from 8 AM to 8 PM, allowing us to explore temporal dynamics of affect and behavior, and how this relates to hikikomori symptoms.

The study proposes the first research question (RQ1): What factors are related to hikikomori symptoms? The first hypothesis is that “hikikomori symptoms will be positively correlated with internet addiction, social anxiety, expressive suppression, and fear of offending others as measured at baseline” (H1a). The second hypothesis is that “hikikomori symptoms postmonitoring period will be predicted by averaged daily internet usage, sleep quality, sleep duration, physical activity, familial contact, anticipatory and consummatory social enjoyment, mood, and emotional variability, while controlling for depression, autism, and anxiety” (H1b).

Additionally, we will explore the temporal dynamics of momentary mood, internet and familial behaviors, and how these differ in individuals with high and low symptoms of hikikomori. This study also proposes the second research question (RQ2): How do internet, social, and familial behaviors predict future social behavior and enjoyment? The first hypothesis is that “recent social enjoyment will predict the likelihood of future social interaction” (H2a). The second hypothesis is that “recent internet usage will predict the likelihood of future social interaction” (H2b). The third hypothesis is that “recent familial support will predict the likelihood of future social interaction” (H2c). The final hypothesis is that “Recent mood and anger will predict the likelihood of future social interaction” (H2d).

## Methods

### Study Design

This study will be a longitudinal observational design, using EMA over a 2-week period. An Adapted STROBE Checklist for Reporting EMA Studies (CREMAS) is included in Checklist 1.

### Transparency and Openness Statement

Anonymized data and analysis code will be made available from the OSF page for this project [33]. The first draft of the protocol paper was submitted before data collection commenced. The study aims, hypotheses, and analysis plan were pre-registered after data collection commenced, with 15 participants recruited at the point of preregistration [34]. No data had been accessed at the point of pre-registration.

### Ethical Considerations

This study received approval from the ethics committee for Life Sciences and Medical Experiments at the National Institute of Advanced Industrial Science and Technology, Japan (S2025-0595). Participants will be fully informed of the study aims prior to giving their consent to participate and are paid a total of 21,000 JPY (US $136.10) for their participation in the wider project, of which this study is one part. Data are anonymous at the point of collection.

### Measures

*Hikikomori symptoms* will be measured at baseline and postmonitoring period, with the 1-month version of the Hikikomori Questionnaire (HQ-25M) [35]. Participants rate their endorsement of statements about social withdrawal symptoms over the last month, and this has been validated in preclinical samples [35]. Items are rated on a 0 (“Strongly Disagree”) to 4 (“Strongly Agree”) scale, with item scores totaled so that higher scores indicate greater severity of hikikomori symptoms. This scale was developed and validated in nonclinical samples, showing positive correlations with both withdrawal behavior and psychological distress at withdrawal [35], and was considered appropriate for the nonclinical sample in this study.

*Depressive symptoms* will be measured with the Japanese version of the Patient Health Questionnaire-9 [36]. Participants rate the frequency of depressive symptoms experienced over the previous 2 weeks on a 0 (“Not at all”) to 3 (“Almost every day”) rating scale. Item responses are totaled with higher scores indicating greater depressive symptom severity. The Japanese version of the Patient Health Questionnaire-9 has been found to be in accordance with neuropsychiatric interviews [36] and suitable for screening and monitoring symptoms in primary care populations [37].

*General anxiety symptoms* will be measured with the Japanese version of the Generalized Anxiety Disorders Scale [38]. On 7 items, participants rated the frequency of anxiety symptoms from 0 (“Not at all”) to 3 (“Every Day”). Item responses are totaled, so that higher scores indicate higher frequency of anxious symptoms. The Japanese Language scale has been validated in clinical and nonclinical samples [38].

*Social anxiety symptoms* will be measured with the Japanese version of the Liebowitz Social Anxiety Scale [39]. This is a 24-item scale on which participants rate their fear of social situations from 0 (“None”) to 3 (“Severe”), and how frequently they avoid those situations from 0 (“Never, 0% of the time”) to 3 (“Usually, 67%‐100% of the time). All item responses are summed, with higher scores indicating greater social anxiety. The Japanese version of the scale has been repeatedly investigated and validated in both clinical and nonclinical groups [39,40].

*Fear of offending others* will be measured with the Taijin Kyofusho Scale [41]. This scale is designed to capture a culturally specific form of social anxiety centered on the fear of offending others with one’s appearance or behavior. Participants rate their agreements with 31 statements from 1 (“Totally Disagree”) to 7 (“Totally Agree”). Item responses are totaled so higher scores indicate higher levels of social anxiety and fear of offending others. The scale has been validated in clinical and nonclinical populations [41,42].

*Internet addiction* will be measured with the Japanese version of the Internet Addiction Test. Participants rate the frequency of internet addiction symptoms on a 0 (“Not at all”) to 5 (“Always”) rating scale. Item responses are totaled so that higher scores indicate more problematic internet use. The scale has been validated in Japanese populations [43] and has been frequently used in research examining the behavior of hikikomori [8].

*Autism traits* will be measured with the Japanese version of the Autism Spectrum Quotient short form [44]. Participants rate their agreement with statements consistent with autistic traits from “Strongly Disagree” to “Strongly Agree.” Answers consistent with autistic traits, for example,“I find it difficult to understand people’s intentions” rated as “Agree,” are given a score of 1. Item responses are totaled, with higher scores indicating greater presence of autistic traits. The scale has been validated in clinical [44] and student samples [45].

*Emotion regulation style* will be measured with the Japanese version of the Emotion Regulation Questionnaire [46]. Participants rate their agreement with 10 statements about emotional regulation strategies (Suppression and Reappraisal) on a 1 (“Strongly Disagree”) to 7 (“Strongly Agree”) rating scale. Scores for each subscale are totaled, with greater scores indicating greater use of that strategy. The scale has been validated in Japanese samples and against measures of personality, well-being, and emotion experience [46].

### EMA Measurements

*Mood and emotional variability* will be estimated from participant ratings on questions about their general mood (“Very negative” to “Very positive”) and how anxious, irritable, sad, and happy (“Not at all” to “Very”) they are at that moment on a 0 to 100 visual analog scale (VAS). Additionally, participants will be asked if they have been angry since the last beep and if so, how angry they were on a 0 to 100 VAS scale (“Not at all” to “Very”). From each mood item rating, we will calculate emotional variability as the within-person SD. Emotional stability will be calculated as the mean squared successive difference between scores. Emotional inertia will be calculated as the autocorrelation of moods across time.

Daily emotional variability will be calculated for each emotion as the SD of that emotion’s ratings each day, while the overall emotional variability will be the mean of that emotion’s daily emotional variability. Overall emotion rating will be calculated as the mean of that emotion’s rating across the assessment period.

*Anticipatory and consummatory social enjoyment* will be assessed using momentary surveys. In the morning survey, participants will be asked to name an event they are looking forward to that day (if one exists), and to rate how much they expect to enjoy it on a 0 to 100 VAS scale (“Not at all” to “Very much”). In the subsequent surveys, participants will be asked whether they have had their social contact since the last survey, whether this was online or in person, how much they enjoyed it (“Not at all” to “Very much,” 0‐100), and whether they desire social contact again that day (or the next day in the evening survey). The enjoyment prediction gap will be calculated as the difference between the anticipated enjoyment rating of an event that day and the consummatory enjoyment rating of the beep after that event occurred.

*Sleep quality and physical activity* will be estimated from a self-report question about sleep quality in the morning survey (“Very bad” to “Very Good,” 0‐100), what time they went to bed and what time they woke up. Physical activity level will be estimated via step counter and fitness tracker, sleep patterns, duration, and quality will be estimated from the fitness tracker and supported by self-report measures.

*Internet and smartphone use* will be estimated from participants’ self-reports on the number of minutes spent using their phone, how many minutes they have spent browsing apps, and how many minutes they have spent using the internet in the 2 hours prior to the survey. Participants will also be asked for what purposes they have used the internet and smartphone apps in that time (social networking, ordering food or other life management, gaming, shopping, to watch videos, to distract them from boredom).

*Familial support* questions will ask about the number of interactions with family members since the last notification and the nature of those interactions. Given the first author is not fluent in Japanese, text responses will be translated into English via translation software for coding. To check for accuracy, 10% of the events will be translated back into Japanese, and their codes will be shared with a native Japanese collaborator to check for accuracy. Coding will be both inductive and deductive, as events will either be classified as enabling or challenging a social isolation, but categories will be derived from the data. Textual responses will not be analyzed further if the context is not about social withdrawal. Each event will be assigned to one of the support styles (enabling vs challenging isolation) for statistical analysis.

### Recruitment Methods

We will recruit a minimum of 84 participants to this study, as part of a wider project being advertised through the University of Tsukuba’s online research recruitment portal, and professional research companies. Participants will be allocated to either “high” or “low” hikikomori symptom groups based on their responses to the HQ-25M, with those scoring ≥42 at baseline being deemed “at risk” of hikikomori, based on cutoffs established in the original scale development paper [47]. This score has been deemed to have “excellent” diagnostic accuracy, sensitivity, and positive predictive value in the original 6-month scale [35]. As items are identical between the original scale and the 1-month version, this potentially represents a valid cutoff in this study and has been used in previous research with the HQ25-M [48]. However, as an HQ25-M cutoff is yet to be validated, we will additionally run sensitivity analyses with a cutoff set as the mean HQ25-M score +1SD, consistent with previous research [49]. Should our primary recruitment method result in a marked imbalance in group numbers after 30 participants have been recruited, we will also recruit participants online via social media and professional recruitment sites. Participants recruited online who do not meet the necessary group allocation criteria (eg, scoring ≥42 on the HQ-25M if the “high” symptom group is lacking participants) will not be eligible to take part. Participants who are eligible to take part will continue to complete the rest of the baseline measures and will be given instructions on how to download the EMA platform on their smartphone. Participants are eligible for the study if they are between the ages of 18 and 60 years, have a smartphone and a stable internet connection, and can stand up independently. Exclusion criteria for the overarching project are those: (1) affected by cardiac, neurological, cerebrovascular, renal, respiratory, or endocrine diseases; (2) currently receiving counseling or psychiatric medication; (3) who cannot exercise due to illness; (4) who work regular night shifts or irregular working hours; (5) who take drugs which may affect cortisol response; (6) who smoke; (7) who regularly use nail polish or artificial nails; (8) who are pregnant; and (9) who are experiencing irregular menstruation, amenorrhea, or menopause.

### Procedure

Participants will be invited into the laboratory to complete all self-report measures and receive instructions on how to download and set up the EMA app, mPath (KU Leuven; [50]), with surveys delivered 5 times per day for 14 days. Surveys will be delivered on a fixed schedule, with the morning survey to be delivered at 8 AM; three more to be delivered at 11 AM, 2 PM, and 5 PM; and the evening survey at 8 PM. There will be a random margin of 15 minutes for the delivery of each prompt (eg, the 11 AM survey may be delivered between 11 AM and 11:15 AM). Participants have a 45-minute response window before that survey is closed and recorded as a missed response. Participants will be nudged to respond if a response has not been recorded within 15 minutes of the first notification being delivered. After the 2-week monitoring period, participants will complete the HQ-25M again.

### Statistical Analysis

#### Sample Size Justification

Given the lack of previous research, our sample size was determined by the resources available for this project, rather than on the basis of previously reported effect sizes. Therefore, we conducted sensitivity power analyses for each of our confirmatory hypothesis tests to determine which effect sizes could be detected with 90% power at our minimum target sample size of 84. With this sample, we will be able to detect effects equivalent to *r*=0.34 in a correlational analysis for H1a, *f^2^*=0.31 for H1b, and pseudo-*R^2^* approximately 0.21 to 0.42 for H2a to H2d. Given that these effect sizes are comparable to those found in related populations such as those with social anxiety (eg, correlation between social anxiety and perseverative thinking *r*=0.55 [51]; difference between hikikomori and controls on depressive symptoms, standardized mean difference=0.78; [52]), we believe this study will be informative to the wider literature on social withdrawal, as well as on hikikomori specifically [53]. Our target of 84 participants completing EMA measures at 5 time points across the 2-week data collection period would result in 70 data points per participant, and a maximum of 5880 data points.

#### Data Exclusion

We will check and report compliance rates for participants. We will include all participants in our primary analyses, though we will conduct separate sensitivity analyses excluding participants who complete less than 75% of the EMA measures, reporting the results in Multimedia Appendix 1. Additionally, missing data will be assessed for randomness via logistic regression. Each of our baseline and demographic variables will be entered into a multiple logistic regression model to determine whether they influence nonresponse.

#### Analysis Plan

##### Overview

Significance will be accepted when *P*≤.05, after the Benjamini-Hochberg false detection rate (FDR) corrections have been applied within the families of hypothesis tests (eg, H1b, H2a, and so on). For exploratory analyses, no significance threshold is set, but *P* values will be interpreted alongside effect sizes and CIs [54] to determine evidence of an effect of interest for future research.

##### RQ1: What Factors are Related to Hikikomori Symptoms?

H1a will be tested with the Pearson correlation analyses, correlating baseline hikikomori symptoms with measures of social anxiety, expressive suppression, cognitive reappraisal, and fear of offending others.

H1b will be assessed with multiple linear regression. Postmonitoring period hikikomori symptoms will be regressed on averaged daily internet usage, averaged sleep quality, averaged sleep duration, averaged physical activity, averaged familial support, averaged anticipatory and consummatory social enjoyment, averaged mood ratings, and overall emotional variability. Symptoms of depression, autism, anxiety, and hikikomori at baseline will be entered as covariates in this model. The hypothesis will be confirmed by the overall model significance and fit, while individual predictor contributions will be assessed by standardized coefficients, FDR corrected *P* values, and CIs. Exploratory analyses will be conducted with pre-hikikomori symptoms and averaged hikikomori symptoms as the outcome, respectively.

To address our exploratory aim, we will construct time-lagged network analyses. For each hikikomori group, multilevel vector autoregressive models (mlVARs) will be specified for each momentary variable [55,56]. The models will be constructed with 2 levels: within day (level 1) and nested within participants (level 2). Momentary mood, anger, irritability, sadness, happiness, consummatory enjoyment, internet use, and family contact at time *t–*1 will be entered as our level 1 predictors. The mlVAR model will be estimated using maximum likelihood estimation and taking the form:


$$Mood_{t}=\;\beta_{0}+\;\beta_{1}Mood_{t-1}+\;\sum_{k=2}^{7}\beta_{k}Anger_{t-1}+\gamma_{1}Anger_{t}+\sum_{k=2}^{6}\gamma_{k}Irritable_{t}+\varepsilon_{t}+\left(1|participant\right)$$

where β_0_ is the intercept, β*_k_* is the coefficient for a variable *k* at the lagged time point *t*–1, γ*_k_* is the coefficient for a variable *k* at the current time point *t*, and ε*_t_* is the error term. Coefficients and SEs will be drawn from these models to construct the time-lagged network [56]. Nodes in the model will represent the variables, while edges will represent cross-lagged and contemporaneous relationships.

Edge weights and centrality measures will be compared between groups with 2-tailed *t* tests, though given the lack of previous research, we will not report these results as confirmatory hypothesis tests. Rather, these results will be interpreted to develop a theory and guide future research efforts.

##### RQ2: How do Internet, Social, and Familial Behaviors Predict Future Social Behavior and Enjoyment?

The hypotheses for this research question will be addressed with four multilevel autoregressive logistic regressions:

1. H2a predictors: consummatory pleasure rating at *t–1*
2. H2b predictors: internet time and usage reason at *t–1*
3. H2c predictors: familial support amount and style (enabling vs challenging) at *t–1*
4. H2d predictors: mood and anger ratings at *t–1*

Regression models will additionally include age, gender, and employment status as covariates, and baseline hikikomori symptoms as moderators. Hikikomori symptom scores will be mean-centered, and significant interaction terms will be examined via a simple slopes analysis. For example, the model for H2b will be:


$$logit\left(P\left(Social\;Encounter_{it}\right)\right)=\beta_{0}+\beta_{1}TimeOnline_{it-1}+\sum_{k=2}^{8}\beta_{k}ReasonOnline_{it-1}^{\left(k\right)}+\beta_{9}SocialEncounter_{it-1}+\beta_{10}HikikomoriScore_{i}+\;\beta_{11}\left(TimeOnline_{it-1}*HikikomoriScore_{i}\right)+\;\sum_{k=12}^{18}\beta_{k}\left(ReasonOnline_{it-1}*\;HikikomoriScore_{i}\right)+\;\beta_{19}\left(SocialEncounter_{it-1}*HikikomoriScore_{i}\right)+\varepsilon_{i}$$

where β_0_ is the intercept, β*_k_* is the coefficient for a variable *k* at the lagged time point *t*–1, *i* represents the participant, and ε*_i_* is the error term. Hypotheses will be confirmed by overall model significance and fit, while the individual predictor contributions will be assessed by odds ratios, FDR corrected *P* values, and CIs.

### Exploratory Analyses

We will conduct exploratory multilevel logistic regressions to determine if averaged daily mood, internet use, consummatory enjoyment, and familial support predict future social event intention in the evening survey.

To investigate whether hikikomori symptoms are related to social reward feedback, we will explore whether social enjoyment prediction gap at *d*–1 is related to the change in anticipatory pleasure rating on the morning survey of the current day *d* from the previous day *d*–1, and whether hikikomori symptoms moderate this relationship.

## Results

This project was funded in May 2025. Data collection began on November 21, 2025, with 15 participants enrolled at the point of preregistration. Results of the study are expected to be available by the end of summer 2026, and no data have been accessed at the time of this paper’s submission.

## Discussion

### Interpretations and Implications of Results

The results of RQ1 will allow us to identify which factors may be most influential in hikikomori. First, the results of our regression will indicate which factors relate to, and predict, hikikomori symptoms after controlling for the symptoms of depression, anxiety, and autism.

By conducting network analyses in high and low symptom groups and comparing the centrality and edge weights, we will be able to identify influential factors in hikikomori, as well as understand how the strength and structure of networks differ at low and high levels of symptoms, which could have implications when developing new treatments.

The results of RQ2 will allow us to identify a candidate mechanism underlying hikikomori and identify maintenance factors for intervention. Results from the model predicting social enjoyment have implications when positioning hikikomori as a consequence of social anhedonia. Should social enjoyment interact with hikikomori symptoms and predict future social behavior (H2a), this would suggest that social anhedonia is one mechanism underlying withdrawal. This position will be further explored with our exploratory analyses investigating whether the social enjoyment–prediction gap update size predicts change in anticipatory pleasure ratings. A null result in this model could suggest a lack of reinforcement learning from social contexts, indicating that reward processing both at the anticipatory and learning stages is impaired, as in depression [21]. A significant result here would suggest normal learning from a social context.

Subsequent models (H2b-H2d) would identify maintenance factors of hikikomori, as well as suggest targets for cognitive or behavioral intervention. These models are expected to be significant given prior correlational research [1,23]; however, this study will be the first to explore these relationships longitudinally. Should null results be found, this would be unexpected but would have implications for current treatment strategies.

In summary, RQ1 explores the relationship between previously identified psychological and social risk factors and self-reported hikikomori traits, while controlling for mental health symptoms frequently associated with hikikomori and hikikomori risk factors. RQ2 investigates the temporal dynamics of these relationships, seeking to validate previously suggested relationships (eg, between internet use and social withdrawal), and mechanisms proposed in other disorders that have yet to be explored in relation to hikikomori (eg, social consummatory pleasure). Taken together, the results of this study will identify important psychological variables that may underlie hikikomori traits and behaviors that may be targeted for early intervention.

### Limitations

The study has several limitations. First, though we are drawing inferences based on symptoms of hikikomori, we are not recruiting a clinical population for this study, and so results may not generalize to clinical samples. For example, given that the isolation in clinical samples will be more marked and prevalent, it may be that the associations found in this study, especially when examining RQ2, are not replicated. This decision was made for practical reasons, and we will draw conclusions in line with this limitation, specifically how these results may be most applicable to those “at risk” or pre-hikikomori. Additionally, we will propose future work in clinical samples. Second, though items are identical between the original scale and 1-month version of the scale, our chosen cutoff score has not been validated. Further psychometric investigation of the HQ25-M is required to determine if the cutoff of 42 is valid. Third, there are problems inherent in interpreting causation in our time-varying models given that we are establishing a Granger causality rather than causality through experimental manipulation. In addition, our analysis plan specifies an mlVAR model where time is treated as a discrete variable and assumes that measurements are taken at equidistant intervals, which is unlikely given the laxity in both survey delivery and response windows and so may bias estimates [56]. We also have specified a 1-step lag in our models, which may exclude important connections at longer lags [57].

We also must acknowledge the modest target sample size for this study, which is primarily driven by time and resource constraints. Though we have attempted to lend some context on what effect sizes may be detectable, given the lack of previous work using EMA methods or our planned analyses, we were not able to conduct either an *a priori* power analysis to inform a target sample size, nor offer more specific potential effect sizes. We acknowledge the risk of a moderate sample size reducing the positive predictive value of our findings [58], though again reiterate the value in this study both in generating new avenues of future research and in testing confirmatory hypotheses based on previous literature. Finally, though a strength of this study is the EMA methodology, we are not including several passive sensing measures in our protocol (eg, location tracking, app and phone usage data, and so on). These data can be collected without any additional participant burden and have been found to predict episodes of mood disorders (eg, depression [59]), though this evidence is mixed in studies investigating social isolation [60] and both the privacy and ethical concerns must be carefully managed in such studies.

### Conclusions

This will be, to our knowledge, the first study investigating the temporal dynamics of proposed maintenance factors of hikikomori and social behavior. Additionally, this study aims to identify influential cognitive, affective, and behavioral factors underlying hikikomori. These results will test and validate previously identified factors from case studies and cross-sectional work and open up avenues for future research in clinical or general samples.

## Supplementary material

10.2196/81384 Multimedia Appendix 1 Revised sample size estimation and ecological momentary assessment (EMA) study reporting guidelines.

10.2196/81384 Checklist 1 CREMAS reporting checklist.

## Abbreviations

EMA: ecological momentary assessment
FDR: false detection rate
HQ-25M: Hikikomori Questionnaire, 1-month version
RQ: research question
VAS: visual analog scale
