# Supplementary material for: Exploring the Cognitive and Behavioral Risks and Maintenance Factors of Hikikomori: Protocol for an Ecological Momentary Assessment Study
Source: JMIR Res Protoc. 2026 Feb 17;15:e81384. doi: 10.2196/81384 (PMC12912654; doi:10.2196/81384)
Supplement: Multimedia Appendix 1 [file resprot-v15-e81384-s001.docx]

### **Sensitivity power analysis R code and output with comments**

> # H1a correlation effect size @ 90% power (each correlation before correction)

> pwr.r.test(n = 84, sig.level = 0.05, power = 0.90,

+ alternative = "greater") # greater as directional hypotheses

approximate correlation power calculation (arctangh transformation)

n = 84

r = 0.3124099

sig.level = 0.05

power = 0.9

alternative = greater

>

> # H1b regression overall effect size @ 90% power. Predictors are: 1) daily internet usage, 2) averaged sleep quality, 3) averaged sleep duration, 4) averaged physical activity, 5) averaged familial support, 6) averaged anticipatory and 7) consummatory social enjoyment, 8) averaged mood ratings, 9) overall emotional variability, 10) symptoms of depression, 11) autism, 12) anxiety and 13) hikikomori at baseline

>

> H1b_power <- pwr.f2.test(u = 13, v = 70, sig.level = 0.05, power = 0.90)

> H1b_power

Multiple regression power calculation

u = 13

v = 70

f2 = 0.3110064

sig.level = 0.05

power = 0.9

> H1bf2 <- H1b_power$f2

> H1bR2 <- H1bf2 / (1 + H1bf2)

> H1bR2

[1] 0.2372272

>

> # H2 regression overall effect sizes @ 90% power based on fewest (H2a) to most (H2b) number of predictors. As we are equating a psuedo R2 and R2 from linear regression, these effect sizes are approximate and will be supported in the main paper by informed power analyses.

>

> H2a_power <- pwr.f2.test(u = 5, v = 77, sig.level = 0.05, power = 0.90)

> H2a_power

Multiple regression power calculation

u = 5

v = 77

f2 = 0.2131495

sig.level = 0.05

power = 0.9

> H2af2 <- H2a_power$f2

> H2aR2 <- H2af2 / (1 + H2af2)

> H2aR2

[1] 0.1756993

>

> H2b_power <- pwr.f2.test(u = 24, v = 61, sig.level = 0.05, power = 0.90)

> H2b_power

Multiple regression power calculation

u = 24

v = 61

f2 = 0.4232064

sig.level = 0.05

power = 0.9

> H2bf2 <- H2b_power$f2

> H2bR2 <- H2bf2 / (1 + H2bf2)

> H2bR2

[1] 0.2973612
